# Adult Participation in Aerobic and Muscle-Strengthening Physical Activities — United States, 2011

**Published:** 2013-05-03

**Authors:** Carmen D. Harris, Kathleen B. Watson, Susan A. Carlson, Janet E. Fulton, Joan M. Dorn, Laurie Elam-Evans

**Affiliations:** Div of Nutrition, Physical Activity, and Obesity, National Center for Chronic Disease Prevention and Health Promotion; Public Health Surveillance and Informatics Program Office, Office of Surveillance, Epidemiology, and Laboratory Services, CDC

The *2008 Physical Activity Guidelines for Americans* states that aerobic and muscle-strengthening physical activities provide substantial health benefits for adults (1). To assess participation in aerobic physical and muscle-strengthening activities among adults in the United States, the Behavioral Risk Factor Surveillance System (BRFSS) included new questions in 2011.* CDC analyzed the 2011 BRFSS survey data for U.S. states and the District of Columbia (DC) and found that the self-reported activities of 20.6% of adult respondents met both aerobic and muscle-strengthening guidelines. Among U.S. states and DC, the prevalence of adults meeting both aerobic and muscle-strengthening guidelines ranged from 12.7% to 27.3%. Nationwide, 51.6% of U.S. adults met the aerobic activity guideline, and 29.3% met the muscle-strengthening guideline. State public health officials can use these data to establish new baselines for measuring progress toward meeting the physical activity guidelines.

BRFSS is a state-based, random-digit–dialed telephone survey of the noninstitutionalized U.S. civilian population aged ≥18 years. Data for the 2011 BRFSS survey were collected from 497,967 respondents and reported by the 50 states and DC. Response rates were calculated using standards set by the American Association of Public Opinion Research.† The response rate is the number of respondents who completed the survey as a proportion of all eligible and likely eligible persons. The median survey response rate for combined landline and cellular telephone respondents for all states and DC in 2011 was 49.7% (range: 33.8%–64.1%).

The assessment of the aerobic activity guideline excluded 39,879 respondents because of missing information, leaving 458,088 usable responses, and the assessment of the muscle-strengthening guideline excluded 28,655 respondents for the same reason, leaving 469,312 usable responses. The assessment of the proportions of persons meeting both the aerobic and muscle-strengthening guidelines excluded 44,246 respondents with missing physical activity data, leaving 453,721 usable responses. Persons with missing educational attainment or body mass index (BMI) data were excluded from education and BMI analyses.

In 2011, to assess participation in aerobic physical activity, respondents were asked to report the frequency and duration of the two aerobic physical activities, outside of regular job duties, at which they spent the most time during the past month or week. To assess participation in muscle-strengthening activities, respondents were asked to report the frequency of their participation in activities to strengthen their muscles during the past month or week. Minutes of activity per month were converted into minutes of activity per week by dividing monthly minutes by the number of weeks in a month. Respondents were classified as meeting both the aerobic and muscle-strengthening guidelines if they met 1) the aerobic activity guideline (≥150 minutes per week of moderate-intensity aerobic activity, or ≥75 minutes of vigorous-intensity aerobic activity, or an equivalent combination of moderate- and vigorous-intensity aerobic activity [where vigorous-intensity minutes are multiplied by 2] totaling ≥150 minutes per week) and 2) the muscle-strengthening guideline (muscle-strengthening activities at least two times per week) (1).

To count toward meeting the aerobic activity guideline, activities had to be classified as aerobic and had to be performed for ≥10 minutes per episode (2). Consistent with earlier (1984–2000) BRFSS classification of aerobic intensity for specific physical activities (3,4), the cut point for defining vigorous-intensity activities in the 2011 BRFSS was ≥60% of a respondent’s estimated aerobic capacity, based on age and sex (3). Moderate-intensity activities were defined as activities using ≥3.0 metabolic equivalents§ and less than the respondent’s vigorous-intensity cut point (2,3). Data were analyzed by demographic characteristics and weighted to provide prevalence estimates; 95% confidence intervals were calculated for each estimate. Orthogonal polynomial contrasts and pairwise t-tests were used to identify significant trends and differences by subgroups.

For 2011, 20.6% of U.S. adults were classified as meeting both the aerobic and muscle-strengthening guidelines, including 23.4% of men and 17.9% of women (Table 1). By age group, the prevalence of meeting both aerobic and muscle-strengthening guidelines ranged from 30.7% among persons aged 18–24 years to 15.9% among those aged ≥65 years. Among racial/ethnic groups, prevalence was lower among Hispanic adults (18.4%) than among non-Hispanic blacks (21.2%) (p<0.001) and non-Hispanic whites (20.7%) (p<0.001). By education level, college graduates had the highest prevalence of adults meeting both aerobic and muscle-strengthening guidelines (27.4%); this decreased by decreasing education levels, with persons who had less than a high school diploma having the lowest prevalence (12.0%). By BMI, prevalence was lower for obese persons (13.5%) than for overweight (21.9%) and underweight/normal weight persons (25.8%). The negative linear relationships between age and meeting both aerobic and muscle-strengthening guidelines and between BMI and meeting the guidelines were both significant (p<0.001), as was the positive linear relationship with education.

Among the 50 states and DC, the prevalence of adults meeting both aerobic and muscle-strengthening guidelines ranged from 12.7% in West Virginia and Tennessee to 27.3% in Colorado (Table 2, Figure). Compared with the South and Midwest, states in the West (23.5%) and Northeast (21.3%) had the highest proportion of adults who met both aerobic and muscle-strengthening guidelines (p<0.001) (Table 1).

Nationwide, 51.6% met the aerobic activity guideline and 29.3% of U.S. adults met the muscle-strengthening guideline (Table 1). Prevalence patterns by sex, education, and BMI for meeting the aerobic activity guideline and the muscle-strengthening guideline were similar to patterns observed for adults who met both the aerobic and muscle-strengthening guidelines combined. Among the 50 states and DC, the prevalence of meeting the aerobic activity guideline ranged from 39.0% in Tennessee to 61.8% in Colorado and for meeting the muscle-strengthening guideline ranged from 20.2% in West Virginia to 36.1% in DC (Table 2).

## Editorial Note

The results of this analysis indicate that approximately one in five U.S. adults met the 2008 guidelines for both aerobic and muscle-strengthening physical activity in 2011. State-based estimates of adults who met both aerobic and muscle-strengthening guidelines ranged from 12.7% to 27.3%. Nationwide, 51.6% of U.S. adults met the aerobic activity guideline and 29.3% met the muscle-strengthening guideline. Within their comparative groups, women, Hispanics, older adults, and obese persons were least likely to have met aerobic and muscle-strengthening guidelines. Additional research is needed to determine the reasons for differences in the proportion of adults who meet aerobic activity guidelines and muscle-strengthening guidelines. The reasons for some states having higher physical activity prevalences have not been explored fully; however, one explanation could be the differences in state demographic distributions (e.g., age, education, or race/ethnicity). For example, states with a higher proportion of non-Hispanic whites (e.g., Oregon: 83.6%, Vermont: 95.3%) had a higher proportion of adults meeting the guidelines than states with a lower proportion of non-Hispanic whites (e.g., Louisiana: 62.6%, Mississippi: 59.1%). However, opportunities exist in all states to increase the proportion of adults participating in aerobic and muscle-strengthening activities.

What is already known on this topic?

Before 2011, state-based prevalences of U.S. adults who met the *2008 Physical Activity Guidelines for Americans* for both aerobic and muscle-strengthening activities were not available. In 2011, the Behavioral Risk Factor Surveillance System (BRFSS) included new questions to assess both of these activities.

What is added by this report?

Based on 2011 BRFSS data, approximately one in five U.S. adults report engaging in enough of both aerobic and muscle-strengthening activities to meet the 2008 guidelines. Among all 50 states and the District of Columbia, the prevalence of meeting both aerobic and muscle-strengthening guidelines ranged from 12.7% to 27.3%. Nationwide, 51.6% of U.S. adults met the aerobic activity guideline, and 29.3% met the muscle-strengthening guideline. Within their comparative groups, lower proportions of women, Hispanics, older adults, and obese persons met the aerobic and muscle-strengthening guidelines.

What are the implications for public health practice?

States that use BRFSS data to set and monitor physical activity goals and objectives can use these new baseline data to track progress toward meeting aerobic and muscle-strengthening guidelines for adults.

The 2011 National Health Interview Survey (NHIS) provides nationally representative data with which to compare findings in this report. Although NHIS and BRFSS use different questions to assess physical activity and different survey methodologies (5), the reported physical activity prevalences are similar. Prevalence estimates were the same in both surveys (20.6%) for meeting both aerobic and muscle-strengthening guidelines (6). For meeting the aerobic activity guideline, prevalence estimates were 48.4% for NHIS and 51.6% for BRFSS; for meeting the muscle-strengthening guideline, prevalence estimates were 24.1% for NHIS and 29.3% for BRFSS.

The 2011 nationwide and state-based prevalence estimates for meeting the aerobic activity guideline differ from previous BRFSS reports (7). In the 2009 BRFSS, the prevalence of persons meeting the aerobic activity guideline was higher (65.4%) than the 2011 BRFSS prevalence described in the current report, and state-based prevalence estimates ranged from 46.7% to 74.3%. These differences are the result, in part, of changes in the BRFSS methods and weighting procedures implemented in 2011 (8) and changes in the questions used to assess aerobic physical activity also implemented in 2011 (4). Because of these changes, data in this report are not directly comparable with data collected from BRFSS before 2011 and set the precedent for new physical activity baseline data. The 2011 data can be used to monitor future physical activity trends using BRFSS.

The findings in this report are subject to at least three limitations. First, BRFSS data are self-reported and might be overestimated because of social-desirability bias, recall limitations, or other factors (9). Second, the median combined landline and cellular telephone response rate was 49.7%, and lower response rates can result in response bias; however, new weighting and survey methodology help to adjust for nonresponse, noncoverage, and undercoverage issues (8). Finally, respondents reported information on their top two physical activities outside of regular job duties. Thus, some respondents classified as not meeting the aerobic guideline criteria might have met the criteria if information about additional aerobic activities or regular, aerobic job duties had been included in the analysis.

Environmental and systems efforts involving communities, schools, governments, and worksites can increase opportunities for physical activity in adults. CDC’s *Guide to Community Preventive Services* recommends eight evidence-based approaches to increase physical activity, including four that address environmental and policy approaches (10). One example is creating or enhancing access to places for physical activity combined with informational outreach. Examples of ways to create opportunities for aerobic and muscle-strengthening activities include establishing joint-use agreements to allow adult use of school facilities during nonschool hours. Other recommended approaches include using street- or community-scale design and practices to provide support and cues (e.g., traffic-calming measures and bicycle amenities) to help adults become more physically active. To implement these approaches, CDC currently funds 25 states to address nutrition, physical activity, obesity, and other chronic diseases by creating supportive environments where persons live, work, learn, and play. CDC’s Community Transformation Grants program also funds activities to improve environments and provide safe, accessible places for physical activity through 61 state and local government agencies, tribes, territories, and nonprofit organizations in 36 states. Continued national, state, and local efforts to implement strategies can help improve the proportion of adults who meet physical activity guidelines.

## Figures and Tables

**FIGURE:**
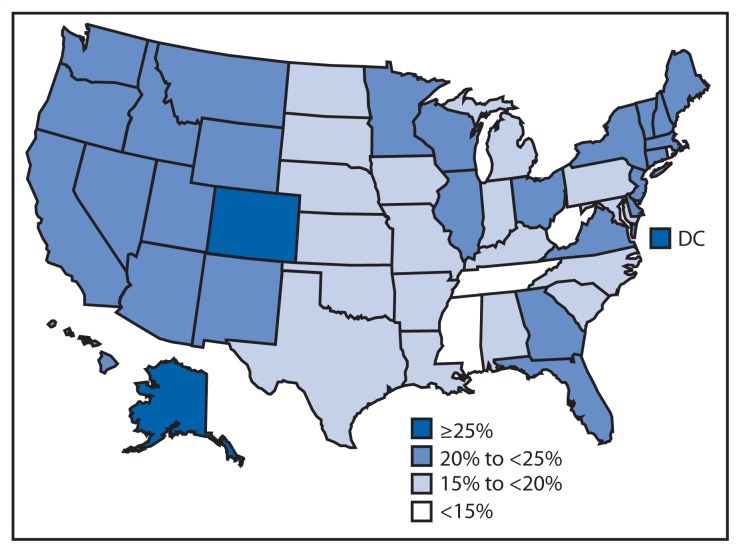
Proportion of U.S. adults meeting both aerobic and muscle-strengthening physical activity guidelines,^*^ by state — Behavioral Risk Factor Surveillance System, United States, 2011 ^*^ To meet both the aerobic and muscle-strengthening guidelines from the 2*008 Physical Activity Guidelines for Americans*, respondents had to report engaging in at least 150 minutes per week of moderate-intensity aerobic physical activity or 75 minutes of vigorous-intensity aerobic physical activity per week, or an equivalent combination of moderate- and vigorous-intensity aerobic physical activity and participating in muscle-strengthening physical activity at least 2 times per week.

**TABLE 1:** Proportion of U.S. adults meeting aerobic and muscle-strengthening physical activity guidelines, by selected characteristics — Behavioral Risk Factor Surveillance System, United States, 2011

Characteristic	Met both aerobic and muscle-strengthening guidelines* (n = 453,721)		Met muscle-strengthening guideline† (n = 469,312)		Met aerobic activity guideline§ (n = 458,088)	
		
	%	(95% CI)	%	(95% CI)	%	(95% CI)
**Total**	**20.6**	**(20.3–20.8)**	**29.3**	**(29.1–29.6)**	**51.6**	**(51.3–51.9)**
**Sex**						
Male	23.4	(23.0–23.8)	34.4	(34.0–34.9)	53.1	(52.6–53.5)
Female	17.9	(17.6–18.2)	24.5	(24.1–24.8)	50.2	(49.8–50.6)
**Age group (yrs)**						
18–24	30.7	(29.7–31.9)	44.1	(42.9–45.2)	56.8	(55.7–58.0)
25–34	23.0	(22.3–23.7)	34.6	(33.7–35.4)	49.8	(49.0–50.7)
35–44	20.4	(19.8–21.0)	29.3	(28.7–30.0)	49.8	(49.0–50.5)
45–54	18.7	(18.2–19.2)	26.1	(25.6–26.7)	51.1	(50.4–51.7)
55–64	17.1	(16.7–17.6)	24.0	(23.5–24.5)	50.9	(50.3–51.5)
≥ 65	15.9	(15.6–16.3)	21.7	(21.3–22.1)	52.7	(52.2–53.2)
**Race/Ethnicity** ¶						
White, non-Hispanic	20.7	(20.4–21.0)	29.0	(28.7–29.3)	53.9	(53.6–54.2)
Black, non-Hispanic	21.2	(20.3–22.2)	31.6	(30.6–32.6)	45.5	(44.5–46.5)
Hispanic	18.4	(17.6–19.3)	27.3	(26.3–28.3)	45.8	(44.7–46.9)
Other race	22.8	(21.6–24.0)	32.9	(31.6–34.2)	51.6	(50.2–52.9)
**Education level**						
Less than high school diploma	12.0	(11.3–12.8)	20.0	(19.2–20.9)	39.2	(38.2–40.2)
High school diploma	17.0	(16.6–17.5)	25.2	(24.6–25.7)	47.5	(46.9–48.1)
Some college	22.2	(21.7–22.7)	31.7	(31.2–32.2)	53.8	(53.2–54.4)
College degree	27.4	(26.9–27.8)	36.6	(36.1–37.0)	60.7	(60.2–61.1)
**Body mass index** **						
Underweight/Normal	25.8	(25.3–26.2)	35.4	(34.9–35.9)	57.0	(56.4–57.5)
Overweight	21.9	(21.5–22.3)	31.0	(30.5–31.5)	54.1	(53.5–54.6)
Obese	13.5	(13.0–13.9)	21.0	(20.5–21.5)	43.4	(42.8–43.9)
**U.S. Census region** ††						
Midwest	20.0	(19.5–20.5)	28.7	(28.2–29.3)	51.3	(50.7–51.9)
Northeast	21.3	(20.7–21.9)	30.0	(29.3–30.6)	52.2	(51.5–52.9)
South	18.7	(18.3–19.2)	27.7	(27.3–28.2)	48.0	(47.5–48.5)
West	23.5	(22.9–24.0)	32.0	(31.4–32.6)	57.2	(56.5–57.8)

**Abbreviation:** CI = confidence interval.

* To meet both the aerobic and muscle-strengthening guidelines from the *2008 Physical Activity Guidelines for Americans,* respondents had to report engaging in at least 150 minutes per week of moderate-intensity aerobic physical activity or 75 minutes of vigorous-intensity aerobic physical activity per week, or an equivalent combination of moderate- and vigorous-intensity aerobic physical activity, and participating in muscle-strengthening physical activity at least two times per week.

† Prevalence of respondents who report participating in muscle-strengthening physical activity at least two times per week.

§ Prevalence of respondents who report engaging in at least 150 minutes per week of moderate-intensity aerobic physical activity or 75 minutes of vigorous-intensity aerobic physical activity per week, or an equivalent combination of moderate- and vigorous-intensity aerobic physical activity.

¶ Other includes multiracial, Asian, Native Hawaiian or Other Pacific Islander, or American Indian/Alaska Native.

** Underweight/normal, overweight, and obese classifications based on body mass index (weight [kg] / height [m]^2^); underweight/normal: <25.0; overweight: 25.0–29.9; and obese: ≥30.0.

†† U.S. Census Bureau regions are defined as *Midwest*: Illinois, Indiana, Iowa, Kansas, Michigan, Minnesota, Missouri, Nebraska, North Dakota, Ohio, South Dakota, and Wisconsin; *Northeast*: Connecticut, Maine, Massachusetts, New Hampshire, New Jersey, New York, Pennsylvania, Rhode Island, and Vermont; *South*: Alabama, Arkansas, Delaware, District of Columbia, Florida, Georgia, Kentucky, Louisiana, Maryland, Mississippi, North Carolina, Oklahoma, South Carolina, Tennessee, Texas, Virginia, and West Virginia; *West*: Alaska, Arizona, California, Colorado, Hawaii, Idaho, Montana, Nevada, New Mexico, Oregon, Utah, Washington, and Wyoming.

**TABLE 2:** Proportion of U.S. adults meeting aerobic and muscle-strengthening physical activity guidelines, by state — Behavioral Risk Factor Surveillance System, United States, 2011

State	Met both aerobic and muscle-strengthening guidelines* (n = 453,721)		Met muscle- strengthening guideline† (n = 469,312)		Met aerobic activity guideline§ (n = 458,088)	
		
	%	(95% CI)	%	(95% CI)	%	(95% CI)
Alabama	15.0	(13.8–16.3)	24.7	(23.3–26.2)	42.4	(40.7–44.0)
Alaska	25.0	(22.8–27.3)	33.8	(31.5–36.3)	57.9	(55.4–60.4)
Arizona	24.2	(22.2–26.3)	32.5	(30.3–34.8)	52.8	(50.4–55.1)
Arkansas	16.7	(14.8–18.8)	24.7	(22.6–26.9)	45.7	(43.3–48.1)
California	23.7	(22.8–24.6)	32.1	(31.1–33.1)	58.2	(57.1–59.2)
Colorado	27.3	(26.1–28.5)	35.6	(34.4–36.9)	61.8	(60.5–63.1)
Connecticut	21.8	(20.3–23.3)	30.6	(29.0–32.3)	52.6	(50.8–54.3)
Delaware	21.5	(19.7–23.4)	32.3	(30.3–34.4)	48.5	(46.4–50.6)
District of Columbia	26.3	(24.2–28.6)	36.1	(33.8–38.5)	57.6	(55.2–59.9)
Florida	21.4	(20.2–22.7)	29.2	(27.8–30.5)	52.8	(51.4–54.3)
Georgia	20.7	(19.4–22.1)	30.2	(28.7–31.8)	50.7	(49.1–52.3)
Hawaii	23.7	(22.2–25.3)	32.1	(30.5–33.8)	58.5	(56.7–60.2)
Idaho	22.4	(20.7–24.2)	30.3	(28.4–32.2)	57.2	(55.2–59.2)
Illinois	22.0	(20.2–23.8)	31.4	(29.5–33.4)	51.7	(49.7–53.7)
Indiana	17.3	(16.0–18.6)	26.0	(24.6–27.4)	46.0	(44.4–47.5)
Iowa	17.2	(16.1–18.5)	27.5	(26.1–28.9)	47.6	(46.1–49.1)
Kansas	16.5	(15.8–17.3)	24.5	(23.7–25.3)	46.8	(45.8–47.7)
Kentucky	17.3	(16.0–18.7)	26.3	(24.8–27.9)	46.8	(45.2–48.5)
Louisiana	15.5	(14.3–16.8)	23.9	(22.6–25.4)	42.0	(40.4–43.5)
Maine	20.6	(19.6–21.6)	27.5	(26.5–28.6)	56.7	(55.5–57.9)
Maryland	19.8	(18.6–21.1)	30.2	(28.8–31.7)	48.7	(47.1–50.2)
Massachusetts	23.3	(22.3–24.3)	32.0	(30.9–33.1)	56.3	(55.1–57.4)
Michigan	19.7	(18.6–20.9)	28.8	(27.5–30.1)	53.5	(52.1–55.0)
Minnesota	20.9	(19.9–21.9)	29.6	(28.5–30.8)	54.0	(52.8–55.2)
Mississippi	14.2	(13.1–15.4)	23.9	(22.5–25.3)	40.0	(38.5–41.5)
Missouri	17.3	(15.9–18.8)	24.7	(23.1–26.3)	49.5	(47.6–51.4)
Montana	21.8	(20.6–23.2)	30.2	(28.8–31.6)	55.3	(53.8–56.8)
Nebraska	19.0	(18.2–19.8)	28.1	(27.3–29.0)	49.0	(48.0–49.9)
Nevada	21.3	(19.3–23.3)	30.1	(27.9–32.4)	52.6	(50.1–55.1)
New Hampshire	22.3	(20.8–23.8)	30.4	(28.8–32.1)	56.1	(54.3–57.8)
New Jersey	23.1	(22.0–24.3)	31.7	(30.5–32.9)	53.2	(52.0–54.5)
New Mexico	22.3	(21.1–23.6)	31.5	(30.2–32.9)	52.2	(50.7–53.6)
New York	21.5	(20.1–23.0)	30.1	(28.6–31.7)	51.5	(49.8–53.1)
North Carolina	18.3	(17.1–19.6)	27.7	(26.3–29.1)	46.8	(45.2–48.3)
North Dakota	18.0	(16.5–19.5)	27.4	(25.7–29.1)	47.3	(45.5–49.2)
Ohio	21.4	(20.1–22.7)	30.4	(29.0–31.8)	51.6	(50.1–53.1)
Oklahoma	16.2	(14.9–17.5)	23.8	(22.4–25.2)	44.8	(43.2–46.3)
Oregon	23.4	(21.9–25.0)	30.9	(29.3–32.6)	61.1	(59.3–62.9)
Pennsylvania	18.8	(17.7–20.0)	27.8	(26.5–29.1)	49.4	(48.0–50.8)
Rhode Island	19.5	(18.1–21.0)	28.5	(26.9–30.2)	48.7	(47.0–50.5)
South Carolina	18.5	(17.4–19.7)	27.6	(26.3–28.9)	50.0	(48.5–51.4)
South Dakota	16.0	(14.5–17.6)	26.1	(24.2–28.1)	46.1	(43.9–48.2)
Tennessee	12.7	(10.7–14.9)	20.6	(18.2–23.2)	39.0	(36.1–41.9)
Texas	19.0	(17.7–20.3)	28.3	(26.9–29.8)	48.2	(46.7–49.8)
Utah	22.5	(21.5–23.6)	32.3	(31.2–33.5)	55.8	(54.6–57.1)
Vermont	21.6	(20.3–23.0)	29.0	(27.6–30.5)	59.2	(57.6–60.8)
Virginia	22.7	(21.1–24.3)	33.4	(31.6–35.3)	52.4	(50.5–54.3)
Washington	21.0	(19.8–22.1)	30.6	(29.3–31.9)	54.2	(52.8–55.6)
West Virginia	12.7	(11.6–14.0)	20.2	(18.8–21.6)	43.0	(41.3–44.7)
Wisconsin	22.3	(20.4–24.2)	29.2	(27.2–31.3)	57.4	(55.2–59.6)
Wyoming	21.2	(19.7–22.8)	29.6	(27.9–31.3)	53.1	(51.3–54.9)

**Abbreviation:** CI = confidence interval.

* To meet both the aerobic and muscle-strengthening guidelines from the *2008 Physical Activity Guidelines for Americans,* respondents had to report engaging in at least 150 minutes per week of moderate-intensity aerobic physical activity or 75 minutes of vigorous-intensity aerobic physical activity per week, or an equivalent combination of moderate- and vigorous-intensity aerobic physical activity and participating in muscle-strengthening physical activity at least two times per week.

† Prevalence of respondents who report participating in muscle-strengthening physical activity at least two times per week.

§ Prevalence of respondents who report engaging in at least 150 minutes per week of moderate-intensity aerobic physical activity or 75 minutes of vigorous-intensity aerobic physical activity per week, or an equivalent combination of moderate- and vigorous-intensity aerobic physical activity.

## Footnotes

* The 2011 BRFSS questions are available at h ttp://www.cdc.gov/brfss/questionnaires.htm.

† Available at http://www.aapor.org/standard_definitions2.htm.

§ One metabolic equivalent is equal to the amount of energy expended while sitting at rest. Additional information and examples are available at https://sites.google.com/site/compendiumofphysicalactivities.
